# Prevalence of menstrual symptoms change and influencing factors among international female students studying in china during acculturation period

**DOI:** 10.1186/s12905-022-01897-6

**Published:** 2022-07-25

**Authors:** Asem Alkhatib, Qidi Zhou, Ousman Bajinka, Ratee Pakwan Suwal, James Wiley, Xianhong Li

**Affiliations:** 1 Xiangya School of Nursing, Central South University, 172 Tong Zi Po Road, Changsha, 410013 Hunan China; 2 Department of Medical Microbiology, Central South University, Changsha, 410078 Hunan China; 3 National Academy of Medical Science, Bir Hospital (NAMS), Mahaboudha, Kathmandu, Nepal; 4 Philip R. Lee Institute for Health Policy Studies, University of California, San Francisco, CA USA

**Keywords:** Menstruation, Symptoms, Changes, Cultural adaptation, International female students

## Abstract

**Background:**

A number of previous studies have explored international students’ adaptation process with regards to language, lifestyle, food, and environment. However, there have yet been no studies conducted to address the menstrual symptoms challenges faced by international female students during the acculturation period. Thus, this study aims to describe the prevalence of menstrual symptoms change and to explore the influencing factors among international female students studying in China during the acculturation period.

**Methods:**

An online cross sectional study was conducted among international female students studying in China during the acculturation period (defined as the first six months of living in the host country) in Hunan Province of China from March 2019 to July 2019. Menstrual symptoms questionnaire, sociocultural adaptation scale, China higher education student satisfaction scale, perceived stress scale, and Pittsburgh sleep quality index were used for data collection. Descriptive analysis, ANOVA, paired *t*-test, Pearson correlation, and multivariate linear regressions were used to analyze the data using SPSS 21.0 software.

**Results:**

Three hundred and forty-five (97.18%, 345/355) female students from 45 countries fully completed the questionnaire. The mean age of the participants was (26.59 ± 6.439) years. In total, 18.49% of participants had encountered menstrual symptoms change. There were significant differences in the menstrual symptoms score between before arrival and evaluation during the first six months of living in China (*t* = − 11.700, *p* = 0.000). The main menstrual symptoms change included cramps (17.68%), irritation (14.78%), abdominal pain (12.46%), fatigue (12.46%), and headaches (9.85%). Cultural adaptation level (β = 0.198, 95% CI: 0.934, 2.995), sleep quality (β = 0.166, 95% CI: 0.112, 0.496), perceived stress (β = 0.193, 95% CI: 0.123, 0.410), time spent in the host environment, (β = − 0.270, 95% CI: − 3.200, − 1.444) and experience of visiting foreign countries (β = 0.184, 95% CI: 1.134, 4.125) were significantly correlated with menstrual symptoms change.

**Conclusion:**

The prevalence of menstrual symptoms change among international female students should not be overlooked when considering menstrual health in this population. Poorer cultural adaptation, poorer sleep quality, higher stress, and lack of overseas living experiences significantly influence the menstrual symptoms of international female students studying in China.

## Background

The menstrual cycle is a normal female reproductive function in which recurrent shedding of blood and tissue from the uterus via the vagina, and its onset is a sign marking puberty in young girls [1, 2]. Maintaining menstrual wellbeing and health is complex and influenced by various psychological, physiological, social, economic, cultural, and other factors [3]. There are several types of menstrual irregularities, including premenstrual syndrome, dysmenorrhea, polymenorrhea, amenorrhea, menorrhagia, oligomenorrhea, but the most prevalent among young females are premenstrual syndrome and dysmenorrhea [4, 5], which refers to symptoms associated with menstruation, including pain, cramp, a change in mood and appetite etc. [6], and may begin a few days before the menstruation and last a few hours to several days [7]. The prevalence of menstrual symptoms ranges between 17% and 90% based on 40 investigations of the general female population worldwide [8, 9]. In addition, many studies reported the prevalence of menstrual symptoms among college students in particular; for instance, it was 88% in Australia [10], 41.7% in China [11], 66% in Egypt [2], 83.6% in Ghana [12], 64% in Mexico [13], 64.85% in Poland [14], 74.8% in Spain [15], and 84% in Turkey [16].

The menstrual symptoms leads to physical or emotional discomfort before or during the menstrual period and this could be considered the main gynecological challenge experienced by adolescents and adult females, and may even have reproductive consequences such as infertility [17], thus poses a considerable burden for females and their families [18]. Menstrual symptoms have a harmful effect on different aspects of female’s lives worldwide, including relationships with family members and friends, performance at school or work, and recreational and social activities [19, 20]. In light of this, females who experienced menstrual symptoms recorded a lower quality of life when compared with women without menstrual symptoms [21].

As for female students, menstrual symptoms could be a factor impacting their rates of absenteeism, performance during classes, and participation in social and emotional activities. This causes a substantial level of psychological distress [22]. In term of the influencing factors, the pattern of menstrual symptoms was reported to be impacted by lifestyle, diet, and environmental factors such as change of weather, nutrition, body weight, and stress [23–25]. In general, international students usually experience the above challenges when pursing higher education in a new environment [26]. They suffered various challenges such as unfamiliar food and living conditions, financial difficulties, work-life imbalance, different study methods, and obstacles linked to the host language and culture [27], and all these changes in life could have strong potential relationships with the menstrual symptoms change [23]. A possible physiological mechanism responsible for menstrual symptoms change among the international female students could be the prolonged activation of the hypothalamic-pituitary-ovarian axis due to cumulative stress level, which may cause hormonal imbalance, resulting in the disruption of normal ovulation and emergence the menstrual symptoms [28, 29]. Understanding the factors which could cause the menstrual symptoms change especially during the acculturation period among international female students may help alleviate the negative effects of students’ experience and improve their productivity in the host environment. However, rare studies were found in the literature to describe the prevalence of menstrual symptoms change among international female students, especially those students studying in China.

With the increasing number of international students studying in China, the psychological and physical health of those students has attracted attention to scholars and educators. A total of 492,185 international students and scholars from 205 countries are studying in China, according to statistics from China’s Ministry of Education in 2018. This makes China one of the most popular destinations in the world for foreign students, and the top choice among Asian destinations [30, 31]. Since China has a long history with a diverse and unique culture, and difficult language, it might make the acculturation period even more challenging for international students. However, less studies were available to describe the different aspects of international students’ adaptation process or experiences to Chinese culture, and the overall wellbeing of these students, especially the menstrual health status remains unexplored [32].

In light of the paucity of information regarding menstrual symptoms among international female students, the main aim of this study is to determine the prevalence of changes in common menstrual symptoms during the acculturation period and to explore the relevant factors involved among international female students studying in China.

## Methods

### Study design

An online cross sectional study was designed with recall for the first six months of living in China and before coming to China to explore the menstrual symptoms change among international female students studying in China during the acculturation period (defined as the first six months of living in the host country). In this study, we defined the menstrual symptoms as the occurred symptoms during the premenstrual and menstrual phases, which mainly represent the premenstrual syndrome and dysmenorrhea.

### Study settings and participants

The target participants were those international female students enrolled in 10 universities located in major cities of Hunan Province, China, since these 10 universities hosted 90% of the international students in Hunan Province [33]. Those female international students who met the following criteria were recruited in the study: aged 18 years and above, studying in Hunan Province, grew up in countries other than China, and understood the English language. Those who were pregnant, having serious mental or physical illnesses (diagnosed gynecological disorders such as primary amenorrhea, polycystic ovary syndrome, and any pathological situations that could cause pathological disturbances of the menstruation), or taking any medication that could impact their menstrual symptoms were excluded.

### Data collection procedure

Data were collected online from March to July 2019 via a Chinese professional survey website (http://www.sojump.com), which could ensure the confidentiality of the data. Initially, with the 10 participating university’s formal permission, flyers were posted on their advertising boards. Flyers were also sent out electronically through WeChat (an instant online chatting platform) groups established by each university for international students. Those who were interested in participating in this study were able to scan the QR code to fill out the questionnaire. Eligible students read the informed consent explaining the purpose, risk, and benefit of the study on the interface of the smart phone or the computer and clicked the “Agree” button, after which they could begin filling out the questionnaire. By submitting the online questionnaire, they were provided with monetary compensation to the value of 10 RMB (1.5 USD) through their WeChat account. The log-in IDs were recorded automatically and thus each smart phone or computer ID could only submit the questionnaire once. Completion of the questionnaire was approximately 20 min in duration, and if the questionnaire was submitted within 8 min, it was deemed invalid. No personal identifiers were collected, and all submitted data was used exclusively for the purposes of the study.

### Variables and measurements

#### Dependent variable

Menstrual symptoms change. The Menstrual Symptoms Questionnaire (MSQ) [34] was used to investigate the female students’ self-reported “recall menstrual symptoms” during the first six months of living in China and before coming to China, the female students were requested to report their menstrual symptoms status retrospectively (for example, Did you feel irritable (easily agitated or impatient) a few days before your period? Before come to China “1 (never) to 5 (always)”, during first six months of living in China “1 (never) to 5 (always)”). The MSQ is a 24-item self-reported measure used to evaluate menstrual symptoms, which was originally developed to assess the dysmenorrhea symptoms, it was widely used to assess the general menstrual symptoms in the literature [35, 36]. The score on each item ranges from 1 (never) to 5 (always), with a higher total score indicating a more severe level of menstrual symptoms. Menstrual symptoms change was calculated by taking scores for menstrual symptoms during the first six months of living in China and subtracting scores for menstrual symptoms before coming to China. The reliability of the subscales of MSQ designated by Cronbach’s alpha ranged from 0.68 to 0.90 [37]. In the current study, the Cronbach’s alpha of the subscale ranged from 0.61 to 0.76 and for the total scale it was 0.90, and the test–retest reliability for the whole scale was 0.82.

#### Independent variables

(1) Sociocultural adaptation. Sociocultural adaptation was measured using the Sociocultural Adaptation Scale (SCAS), a 21-item scale developed by Ward and Kennedy [38]. Participants were asked to answer these items during their first six months of living in China using a 5-point scale ranging from 1 (No difficulty) to 5 (Extreme difficulty). The average score of the 21 items was used to evaluate the total sociocultural adaptation level, with a higher score indicating a greater difficulty in sociocultural adaptation. The Cronbach’s alpha of the SCAS ranged from 0.75 to 0.91, with a mean of 0.85 as reported by a number of past studies [38, 39]. The Cronbach’s alpha of the SCAS in the present study was 0.89, and the test–retest reliability was 0.84.

(2) China higher education student satisfaction. Students’ level of satisfaction was measured using the China Higher Education Student Satisfaction Scale, developed by Liu Hui [40]. This section consisted of three broad statements focusing on whether the students felt satisfied with the quality of education services in the school. Participants were asked to answer these statements during their first six months of living in China, and the responses were recorded on a 10-item Likert scale ranging from (1) strongly dissatisfied to (10) strongly satisfied, with a higher score indicating a higher level of satisfaction. The reliability of the scale, as measured by Cronbach’s alpha, was 0.81 in the original study [40] and 0.83 in the present study, and the test–retest reliability was 0.84 in this study.

(3) Stress. Participants’ psychological stress was assessed using the Perceived Stress Scale (PSS) [41, 42]. The PSS consisted of 10 items, participants were asked to answer these items during their first six months of living in China, and each item is rated on a 5-point Likert scale (0 = *never*, 4 = *very often*), with a higher score reflecting a greater level of perceived stress. The PSS had an internal consistency designated by Cronbach’s alpha of 0.78 [42]. The coefficient of Cronbach’s alpha in our study was 0.67, and the test–retest reliability was 0.76.

(4) The Pittsburgh Sleep Quality Index (PSQI). The PSQI is a self-reported questionnaire that evaluates sleep quality during the previous month [43]. The measure contains 19 individual items, establishing 7 components. Participants were asked to answer these items during their first six months of living in China, and each item is rated from 0 (no difficulty) to 3 (severe difficulty) on the Likert Scale. The global PSQI score is calculated by adding the seven component scores, with a higher value indicating a poorer quality of sleep. The coefficient of Cronbach’s alpha of PSQI was 0.83 [43]. The coefficient of Cronbach’s alpha in our study was 0.72, and the test–retest reliability was 0.79.

#### Covariates

Sociodemographic characteristics. Sociodemographic characteristics mainly included students’ nationality, age, marital status, major, degree program, time living in China, source of funding for education in China, first language, English proficiency, Chinese proficiency, and whether they have been to China or other foreign countries before.

The initial questionnaire including the above scales were developed by the research team, and reviewed by 5 experts in the field to verify the suitability for the objectives of the study. Then the questionnaire was piloted among 30 international female students who were studying in China and met the inclusion criteria to ensure the clarity of all items. And finally the reliability of each scale in this study was reported as above.

### Statistical analysis

Data were analyzed using SPSS 21.0 software (SPSS Inc., Chicago, IL, USA). Frequencies and percentages were used to present categorical variables, and means and standard deviations were used to describe the distributions of continuous variables. We performed an analysis of covariance (ANOVA) to determine the association between sociodemographic characteristics and menstrual symptoms change. The paired *t*-test was used to compare the changes between the time points. Pearson correlation analysis was processed between the independent variables and the dependent variable. Multivariate linear regressions were employed to identify influencing factors for menstrual symptoms change by examining the significant variables during the bivariate analysis.

### Sample size

The sample size was estimated by using the calculation formula to estimate the sample size for prevalence: $n=\frac{u_{a/2}^2\pi(1-\pi)}{\delta^2}$. Since there was no study to describe the prevalence of menstrual symptoms change among college students before, we set π = 0.5 according to the recommendation by the textbook [44] to get the largest estimated sample size, and α = 0.05, and  *δ* = 0.04, then the calculated sample size was 306.

## Results

A total of 355 international female students from 45 countries filled out the questionnaire, and 345 (97.18%) valid questionnaires with complete data were analyzed. The mean age of the participants was (26.59 ± 6.439) years with an age range of 20–49 years. The majority of them were single (75.4%) and were abroad for the first time (71.3%). Approximately half of the participants were undergraduate students (54.8%), and among those, 60% were majoring in medicine (60.3%). Approximately 40% of the students were in the second academic year (enrolled in 2017) at the time of the survey, and 53.3% of the students were beneficiaries of the Chinese Government scholarship. More than the half of students (53.6%) had English as their official language and had a low level of Chinese language efficiency, with 27.5% and 27.8% having passed Hanyu Shuiping Kaoshi (HSK) 3 and 4, respectively. Only 58.6% of them bought health insurance in China. The other sociodemographics characteristics were shown in Table 1.

**Table 1:** General characteristics and sociodemographics of participants and statistical significance factors

Variables	N (%)	Menstrual symptoms change	F value, *p* value
		(Mean, SD)	
Age			
19–28	258 (74.8)	4.28 (6.592)	0.665, 0.515
29–38	65 (18.8)	3.78 (6.820)	
39–48	22 (6.4)	2.73 (3.654)	
Marital status^a^			
Single	260 (75.4)	4.30 (6.590)	0.894, 0.345
Married	82 (23.8)	3.52 (6.244)	
Come from			
Asia	274 (79.4)	4.08 (6.346)	0.169, 0.845
Africa	63 (18.3)	4.29 (7.398)	
Other	8 (2.3)	2.88 (3.357)	
Where living now			
Student dormitory	324 (93.9)	4.17 (6.608)	0.866, 0.353
Renting room outside school	21 (6.1)	2.81 (4.118)	
Major			
Medicine	208 (60.3)	4.33 (6.911)	0.745, 0.389
Non medicine	137 (39.7)	3.72 (5.793)	
Program			
Bachelor	189 (54.8)	4.26 (6.052)	1.236, 0.292
Master	132 (38.2)	4.20 (7.369)	
PhD	24 (7.0)	2.08 (4.053)	
Enrollment year			
2018	111 (32.2)	6.20 (7.971)	13.507, 0.000**
2017	147 (42.6)	4.00 (6.371)	
2016	87 (25.2)	1.54 (2.425)	
Tuition covered by			
Chinese government	184 (53.3)	4.09 (7.005)	0.273, 0.761
Your own country government	7 (2.0)	5.86 (6.719)	
Yourself	154 (44.6)	4.00 (5.834)	
Official language			
English	185 (53.6)	4.52 (6.974)	1.817, 0.179
Others	160 (46.4)	3.58 (5.858)	
Chinese language level			
HSK1	15 (4.3)	1.73 (2.086)	0.778, 0.588
HSK2	8 (2.3)	6.38 (6.435)	
HSK3	95 (27.5)	4.43 (6.668)	
HSK4	96 (27.8)	3.89 (6.619)	
HSK5	34 (9.9)	3.82 (6.767)	
HSK6	8 (2.3)	1.75 (4.559)	
Never took Chinese language level test	89 (25.8)	4.44 (6.688)	
Learned Chinese before coming to China			
Yes	3 (0.9)	5.67 (5.686)	0.179, 0.673
No	342 (99.1)	4.07 (6.500)	
English ability			
Excellent	96 (27.8)	3.63 (6.371)	0.208, 0.934
Very good	95 (27.5)	4.11 (6.176)	
Good	119 (34.5)	4.39 (6.835)	
Fair	29 (8.4)	4.38 (6.763)	
Poor	6 (1.7)	3.67 (6.501)	
English speaking ability			
Excellent	80 (23.2)	3.78 (6.260)	0.344, 0.848
Very good	88 (25.5)	3.68 (5.757)	
Good	123 (35.7)	4.24 (6.845)	
Fair	48 (13.9)	4.79 (6.820)	
Poor	6 (1.7)	5.33 (10.405)	
English reading ability			
Excellent	109 (31.6)	4.39 (6.759)	0.519, 0.721
Very good	96 (27.8)	3.95 (6.267)	
Good	112 (32.5)	4.04 (6.407)	
Fair	21 (6.1)	4.48 (7.554)	
Poor	7 (2.0)	0.86 (2.268)	
English writing ability			
Excellent	92 (26.7)	3.91 (6.379)	0.116, 0.977
Very good	91 (26.4)	4.26 (6.646)	
Good	118 (34.2)	3.92 (6.413)	
Fair	35 (10.1)	4.46 (6.256)	
Poor	9 (2.6)	4.89 (8.950)	
Proportion of classes taught in English			
No English	27 (7.8)	2.81 (4.683)	1.162, 0.324
Less than 50%	49 (14.2)	4.86 (6.792)	
50% and more	197 (57.1)	4.39 (6.938)	
totally English	72 (20.9)	3.21 (5.459)	
Your teacher's English ability			
Excellent	27 (7.8)	3.78 (4.750)	1.050, 0.381
Very good	101 (29.3)	3.46 (6.751)	
Good	155 (44.9)	4.77 (6.834)	
Fair	51 (14.8)	3.16 (4.957)	
Poor	11 (3.2)	5.36 (8.605)	
Been to China before studying here			
Yes	41 (11.9)	0.32 (0.907)	16.412, 0.000**
No	304 (88.1)	4.60 (6.746)	
Been to foreign countries before studying here			
Yes	99 (28.7)	2.21 (5.999)	11.962, 0.001**
No	246 (71.3)	4.84 (6.536)	
Bought any health insurance in China			
Yes	202 (58.6)	3.73 (5.768)	1.455, 0.229
No	143 (41.4)	4.59 (7.379)	

*Menstrual symptoms change* difference of the menstrual symptoms scores during the first six months of living in China and before coming to China, *HSK* Hanyu Shuiping Kaoshi

^a^Total of marital status 342 participants with 1 divorced and 2 widowed

*p < 0.05; **p < 0.01

In total, 18.49% of international female students had encountered menstrual symptoms change (including students who experienced changes in menstrual regularity, students who had never experienced menstrual symptoms before but had symptoms after living in China, and students who had symptoms before coming to China and which became more severe after living in China). The main changes in menstrual symptoms included cramps (17.68%), irritation (14.78%), abdominal pain before and during menstruation (12.46% and 11.3%, respectively), fatigue (12.46%), headaches (9.85%), backaches before and during menstruation (9.27% and 6.95%, respectively), nausea (9.27%), weakness (8.69%), spasm (6.95%), abdominal discomfort (6.95%), and regularity of menstruation (11.8%).

Before coming to China, the average score of menstrual symptoms was 59.49, and during the first six months of living in China, the average score of menstrual symptoms was 63.58. This result indicates a significant difference in menstrual symptoms scores between before arrival and evaluation during the first six months of living in China (*t* = −11.700, *p* = 0.000). A comparison of each item-score between menstrual symptoms before and after arrival in China is illustrated in Table 2.

**Table 2:** The comparison on the score of MSQ before coming to China, the first six months of living in China

Items	MS_BF	MS_F6M	*t* value*, p* value
	Mean ± SD	Mean ± SD	
Feel irritable a few days before the menstruation	2.43 (1.216)	2.85 (1.192)	− 7.912 (0.000**)
Have cramps during the menstruation	2.67 (1.352)	3.13 (1.248)	− 8.571 (0.000**)
Feel depressed for several days before the menstruation	2.41 (1.155)	2.42 (1.149)	− 1.671 (0.096)
Had abdominal pain which begins one day before the menstruation	2.78 (1.377)	3.12 (1.253)	− 7.312 (0.000**)
Feel fatigued for several days before the menstruation	2.57 (1.268)	2.93 (1.230)	− 7.216 (0.000**)
Know that the menstruation coming by looking at the calendar	3.17 (1.439)	3.18 (1.436)	− 1.897 (0.059)
Take a prescription medication for the pain during the menstruation	2.02 (1.241)	2.08 (1.246)	− 3.735 (0.000**)
Feel weak during the menstruation	2.75 (1.227)	2.96 (1.155)	− 5.813 (0.000**)
Feel tense and nervous before the menstruation	2.50 (1.256)	2.61 (1.239)	− 3.871 (0.000**)
Have diarrhea during the menstruation	2.10 (1.226)	2.19 (1.233)	− 3.545 (0.000**)
Have backaches for several days before the menstruation	2.54 (1.359)	2.80 (1.355)	− 6.132 (0.000**)
Take medicine for the pain during the menstruation	2.01 (1.198)	2.07 (1.207)	− 3.101 (0.002**)
Feel that the breasts tenderness a few days before the menstruation	2.66 (1.351)	2.67 (1.345)	− 1.607 (0.109)
Feel any pain in the lower back, abdomen during the menstruation	2.85 (1.389)	3.15 (1.266)	− 6.778 (0.000**)
Heat make you comfortable, such as use a hot water bottle during the menstruation	2.88 (1.430)	2.98 (1.383)	− 4.078 (0.000**)
Gain weight before the menstruation	2.01 (1.207)	2.09 (1.211)	− 3.746 (0.000**)
Had constipation during the menstruation	1.93 (1.087)	2.03 (1.091)	− 4.289 (0.000**)
Have pain spasms during the menstruation	2.42 (1.276)	2.59 (1.261)	− 5.149 (0.000**)
Have dull continuous pain during the menstruation	2.55 (1.200)	2.61 (1.177)	− 2.195 (0.029*)
Have abdominal discomfort more than one day before the menstruation	2.59 (1.298)	2.75 (1.239)	− 4.312 (0.000**)
Have backaches during the menstruation	2.68 (1.310)	2.87 (1.263)	− 5.633 (0.000**)
Feel your abdominal bloated before the menstruation	2.62 (1.309)	2.63 (1.301)	− 1.737 (0.083)
Feel nauseous during the menstruation	2.11 (1.248)	2.35 (1.277)	− 6.125 (0.000**)
Have headaches for a few days before the menstruation	2.24 (1.286)	2.51 (1.299)	− 6.732 (0.000**)
Regularity of the menstruation	1.16 (0.364)	1.28 (0.447)	− 4.889 (0.000**)

*MS_BF* menstrual symptoms before coming to China, *MS_F6M* menstrual symptoms during the first six months of living in China

*p < 0.05; **p < 0.01

The average score of cultural adaptation, education satisfaction, perceived stress, and sleep quality were 2.79, 21.70, 28.31, and 6.78, respectively (Table 3). The differences in menstrual scores were significantly associated with cultural adaptation, perceived stress, and sleep quality (Table 3).

**Table 3:** Description and correlation of the independent factors with menstrual symptoms change

	Range	Minimum	Maximum	Mean	SD	Correlation with menstrual symptoms change
Sociocultural adaptation scale	3.45	1.00	4.45	2.79	0.65	0.198**
China higher education student satisfaction scale	23	7	30	21.70	5.10	− 0.094
Perceived stress scale 10	28	11	39	28.31	4.71	0.193**
Pittsburgh sleep quality index	17	0	17	6.78	3.54	0.166**
Menstrual symptoms change	46	0	46	4.09	6.48	1.00

*Menstrual symptoms change* difference of the menstrual symptoms scores during the first six months of living in China and before coming to China

*p < 0.05; **p < 0.01

The differences in menstrual symptoms were statistically influenced by students’ enrollment year (F = 13.507, *p* = 0.000) and whether they had been to China (F = 16.412, *p* = 0.000) or other foreign countries before (F = 11.962, *p* = 0.001) (Table 1).

All the significant variables during the bivariate analysis were entered into the multivariate linear regression model, and the analysis showed that greater menstrual symptoms change was independently influenced by less time being enrolled (β = − 0.270, 95% CI: − 3.200, − 1.444), never having been to China (β = 0.214, 95% CI: 2.201, 6.355) or other foreign countries before (β = 0.184, 95% CI: 1.134, 4.125), poorer cultural adaptation (β = 0.198, 95% CI: 0.934, 2.995), poorer sleep quality (β = 0.166, 95% CI: 0.112, 0.496), and higher stress (β = 0.193, 95% CI: 0.123, 0.410) (Table 4).

**Table 4:** Multivariate linear regression analysis on the influencing factors of the menstrual symptoms change among international female students in China

Model	Unstandardized coefficients		Standardized coefficients	*t*	*p*	95.0% confidence interval for B	
	B	Std. error	Beta			Lower bound	Upper bound
Enrollment year	− 2.322	0.446	− 0.270	− 5.201	0.000**	− 3.200	− 1.444
Been to China before studying here	4.278	1.056	0.214	4.051	0.000**	2.201	6.355
Been to foreign countries before studying here	2.629	0.760	0.184	3.459	0.001**	1.134	4.125
Sociocultural adaptation	1.965	0.524	0.198	3.749	0.000**	0.934	2.995
China higher education student satisfaction	− 0.120	0.068	− 0.094	− 1.751	0.081	− 0.254	0.015
Perceived stress	0.266	0.073	0.193	3.648	0.000**	0.123	0.410
Pittsburgh sleep quality	0.304	0.097	0.166	3.121	0.002**	0.112	0.496

Menstrual symptoms change: difference of the menstrual symptoms scores during the first six months of living in China and before coming to China

*p < 0.05; **p < 0.01

## Discussion

To the best of our knowledge, this study is the first to describe the challenges regarding menstrual symptoms among international female students in China during the acculturation period. The results of our study showed that a significant percentage of those international female students experienced more frequent and severe menstrual symptoms during the first six months of living in China, and this was influenced by poorer cultural adaptation, poorer sleep quality, and higher levels of stress. The results further highlight the importance of taking adequate care of international female students and paying attention to their menstrual symptoms during the acculturation period, which has typically been neglected.

In the current study, cramp was the most widespread symptom that changed during the acculturation period, as reported by participants. This was consistent with the finding from another study conducted among college female students in China [11]. As culture and food are inseparably linked, changes in individuals’ eating habits or fluctuations in their usual diets could be a reason for muscle cramps, where unnecessary recurrence of cramps happen in motor neurons and muscle sinew impulses, which may lead to muscles shortening and seizing up [45]. Another commonly reported symptom that changed was irritability, a finding which was also reported by another study conducted among international female students in China [23], perhaps as a result of the similarity between the study’s target populations. Furthermore, our study found abdomen pain, back pain, fatigue, and headaches were also frequently reported symptoms that changed. The literature indicates that pain and fatigue were the most frequently reported menstrual symptoms among college students in Turkey [16] and India [46], while headache was reported to be significantly more intense among Spanish college students [47].

The results of the present study confirmed that menstrual symptoms change was significantly correlated with stress, and similar results were reported by Fernández [48] and Mohib [49] who also asserted that a strong association existed between high stress and menstrual symptoms. In addition, a longitudinal study conducted in the US [50] clarified that experiencing high stress in previous months was likely responsible for reports of increased severity and changes in a significant number of symptoms in the following menstruation (premenstrual and menstrual phases). Stress may inhibit the release of luteinizing hormone and follicle-stimulating hormone and thus damage the growth of the follicle. This may in turn change the progesterone production and relief cycle, which could impact the effect of prostaglandin. Additional hormones associated with stress like cortisol and adrenaline may also impact prostaglandin production and may clarify the role of stress in menstrual symptoms [51, 52].

Sleep disturbance was a common issue reported among university students [53], it was particularly common among international female students, and this was due to difficulties adjusting to the host culture, including the dormitory setting and environment [54]. Our study showed that poor sleep quality was linked to the frequency and severity of menstrual symptoms, which inclines us consider that circadian rhythms may play a role in pathophysiology. The literature explains that lack of sleep could lead to hormonal imbalances which are known to disturb the menstrual symptoms. This is attributed to altered melatonin secretion which in turn impacts several hormones such as progesterone, estrogen, growth hormone, and prolactin. These hormones not only control the reproductive tasks but also influence sleep and circadian rhythms [55, 56].

Regarding travel history to China and becoming acquainted with its unique environment and culture, these were considered significant and important factors for better cultural adaptation and in turn better menstrual symptoms. Numerous studies have explored the process of acculturation and patterns of adaptation; however, most of these studies were conducted in environments very different to China [32]. While the Chinese culture remains unique and distinct, and one which is difficult to adapt to compared to other cultures such as the United States and the United Kingdom [57], intercultural sensitivity remains an important factor in facilitating the adaptation to Chinese culture [32].

### Study limitations

A number of limitations were recognized in our study. Recalling the status of menstrual symptoms before arriving in China and previous times in China might result in recall bias. As showed in Table 4, the students who spent a longer period of living in China reported suffering significantly less of the menstrual symptoms change than those who spent a shorter period in China, which might be due to the recall bias. In addition to that, our study’s target population was only in one province of China, which may not represent the experiences of other international female students studying in other parts of China, since the culture differences among different provinces in China are significant. Lastly, inadequate biomarkers were collected to confirm menstrual symptoms in this study as a result of funding limitations. Future studies should therefore be considered to evaluate changes in hormone levels as evidence of menstrual symptoms.

### Study implications

Despite the aforementioned limitations, the results of this study had important implications such as calling attention to the need to take more appropriate care of international female students’ menstrual symptoms, an aspect of international education which has been neglected throughout the world. Assisting international female students with successfully adapting to the host country is essential to ensure the quality of students’ higher education and international reputation of the host country. Therefore, future studies should consider ways to facilitate the cultural adaptation process, improve sleep quality, and reduce perceived stress among international female students, which would not only benefit the cultural adaptation process itself, but would also improve students’ menstrual symptoms and the quality of their international education.

## Conclusion

This study concludes that menstrual symptoms change frequently occur among international female students during the acculturation period. The most common symptoms experienced were cramps, irritation, pain, fatigue, and headaches. The main influencing factors for the menstrual symptoms change were poor cultural adaptation, poor sleep quality, high perceived stress, spending a shorter time in the host environment, and lack of previous experience visiting foreign countries. Since China has become one of the most important destinations in the world for foreign students and the first choice among Asian destinations, more intervention studies focusing on facilitating international female students’ cultural adaptation and improving their menstrual symptoms are needed.

## Data Availability

The datasets generated or analysed during the current study are not publicly available due to concerns about protecting participants’ privacy but are available from the corresponding author on reasonable request.

## Abbreviations

MSQ: Menstrual symptoms questionnaire
SCAS: Sociocultural adaptation scale
PSS: Perceived stress scale
PSQI: Pittsburgh sleep quality index
MS_BF: Menstrual symptoms before coming to China
MS_F6M: Menstrual symptoms during the first six months of living in China
Menstrual symptoms change: Difference of the menstrual symptoms scores during the first six months of living in China and before coming to China
HSK: Hanyu Shuiping Kaoshi

## References

[1] Hennegan J, Sol L (2020). Confidence to manage menstruation at home and at school: findings from a cross-sectional survey of schoolgirls in rural Bangladesh. Cult Health Sex.

[2] Nooh AM, Abdul-Hady A, El-Attar N (2016). Nature and prevalence of menstrual disorders among teenage female students at Zagazig University, Zagazig. Egypt J Pediatr Adoles Gynecol.

[3] Chandra-Mouli V, Patel SV (2017). Mapping the knowledge and understanding of menarche, menstrual hygiene and menstrual health among adolescent girls in low-and middle-income countries. Reprod Health.

[4] Acheampong K, Baffour-Awuah D, Ganu D, Appiah S, Pan X, Kaminga A (2019). Prevalence and predictors of dysmenorrhea, its effect, and coping mechanisms among adolescents in Shai Osudoku District, Ghana. Obstetr Gynecol Int.

[5] Mansoor H, Salman M, Asif N, Mustafa ZU, Nawaz AS, Mohsin J (2020). Menstrual knowledge and practices of Pakistani girls: a multicenter, cross-sectional study. Heliyon.

[6] Alsaleem MA (2018). Dysmenorrhea, associated symptoms, and management among students at King Khalid University, Saudi Arabia: an exploratory study. J Fam Med Prim Care.

[7] Kho KA, Shields JK (2020). Diagnosis and management of primary dysmenorrhea. JAMA.

[8] Kapadi R, Elander J (2020). Pain coping, pain acceptance and analgesic use as predictors of health-related quality of life among women with primary dysmenorrhea. Eur J Obstet Gynecol Reprod Biol.

[9] Mcgovern CE, Corjena C (2018). Yoga and quality of life in women with primary dysmenorrhea: a systematic review. J Midwif Womens Health.

[10] Subasinghe AK, Happo L, Jayasinghe YL, Garland SM, Wark JD (2016). Prevalence and severity of dysmenorrhoea, and management options reported by young Australian women. Aust Fam Phys.

[11] Hu Z, Tang L, Chen L, Kaminga AC, Xu H (2020). Prevalence and risk factors associated with primary dysmenorrhea among Chinese female university students: a cross-sectional study. J Pediatr Adolesc Gynecol.

[12] Ameade EPK, Amalba A, Mohammed BS (2018). Prevalence of dysmenorrhea among University students in Northern Ghana; its impact and management strategies. BMC Womens Health.

[13] Ortiz MI (2010). Primary dysmenorrhea among Mexican university students: prevalence, impact and treatment. Eur J Obstet Gynecol Reprod Biol.

[14] Zurawiecka M, Wronka I (2018). Association of primary dysmenorrhea with anthropometrical and socio-economic factors in Polish university students. J Obstet Gynaecol Res.

[15] Fernández-Martínez E, Onieva-Zafra MD, Parra-Fernández ML (2018). Lifestyle and prevalence of dysmenorrhea among Spanish female university students. PLoS ONE.

[16] Aktaş D (2015). Prevalence and factors affecting dysmenorrhea in female university students: effect on general comfort level. Pain Manag Nurs.

[17] Olowojesiku R, Shim DJ, Moppins B, Park D, Patterson JO, Schoenl SA (2021). Menstrual experience of adolescents in the USA: protocol for a scoping review. BMJ Open.

[18] Nor YA, Nani D, Nor YA, Shamsul AK, Imran A, Shaiful IB (2015). Knowledge of and attitudes towards of menstrual disorders adults in north-eastern state of Peninsular Malaysia. Malays Fam Phys.

[19] Pitangui ACR, Gomes MRdA, Lima AS, Schwingel PA, Albuquerque APdS, de Araújo RC. Menstruation disturbances: prevalence, characteristics, and effects on the activities of daily living among adolescent girls from Brazil. J Pediatr Adoles Gynecol. 2013;26(3):148–52. 10.1016/j.jpag.2012.12.001 23507005

[20] Kirmizigil B, Demiralp C (2020). Effectiveness of functional exercises on pain and sleep quality in patients with primary dysmenorrhea: a randomized clinical trial. Arch Gynecol Obstet.

[21] Söderman L, Edlund M, Marions L (2019). Prevalence and impact of dysmenorrhea in Swedish adolescents. Acta Obstet Gynecol Scand.

[22] Michael J, Iqbal Q, Haider S, Khalid A, Haque N, Ishaq R (2020). Knowledge and practice of adolescent females about menstruation and menstruation hygiene visiting a public healthcare institute of Quetta. Pak BMC Women's Health.

[23] Ansong E, Arhin SK, Cai Y, Xu X, Wu X (2019). Menstrual characteristics, disorders and associated risk factors among female international students in Zhejiang Province, China: a cross-sectional survey. BMC Womens Health.

[24] Bu L, Lai Y, Deng Y, Xiong C, Li F, Li L (2019). Negative mood is associated with diet and dietary antioxidants in university students during the menstrual cycle: a cross-sectional study from Guangzhou, China. Antioxidants.

[25] Rafique N, Al-Sheikh MH (2018). Prevalence of menstrual problems and their association with psychological stress in young female students studying health sciences. Saudi Med J.

[26] Du X, Kariwo M, Asadi N, El-Bouhali C (2019). International students’ daily negotiations in language, culture, and identity in Canadian higher education. Interrogating models of diversity within a multicultural environment.

[27] Yilmaz ND, Sahin H, Nazli A (2020). International medical students’ adaptation to university life in Turkey. Int J Med Educ.

[28] Lee Y, Im EO (2017). Stress and premenstrual symptoms among Korean women studying in the U.S. and South Korea: a longitudinal web-based study. Women Health.

[29] Mikhael S, Punjala-Patel A, Gavrilova-Jordan L (2019). Hypothalamic-pituitary-ovarian axis disorders impacting female fertility. Biomedicines.

[30] Ma J, Zhao K (2018). International student education in China: characteristics, challenges, and future trends. High Educ.

[31] Ministry of Education, National statistics for international students studying in China in 2018. http://en.moe.gov.cn/documents/reports/201904/t20190418_378692.html. Accessed 18 April 2019.

[32] An R, Chiang S-Y (2015). International students' culture learning and cultural adaptation in China. J Multiling Multicult Dev.

[33] Hunan Provincial People's Government, Education Department, Hunan Universities. http://enghunan.gov.cn/hneng/Services/Education/InstitutionsPrograms/index.html. Accessed 30 Dec 2021.

[34] Chesney MA, Tasto DL (1975). The development of the menstrual symptom questionnaire. Behav Res Ther.

[35] Negriff S, Dorn LD, Hillman JB, Huang B (2009). The measurement of menstrual symptoms: factor structure of the menstrual symptom questionnaire in adolescent girls. J Health Psychol.

[36] Beal SJ, Dorn LD, Sucharew HJ, Sontag-Padilla L, Pabst S, Hillman J (2014). Characterizing the longitudinal relations between depressive and menstrual symptoms in adolescent girls. Psychosom Med.

[37] Dorn LD, Negriff S, Huang B, Pabst S, Hillman J, Braverman P (2009). Menstrual symptoms in adolescent girls: association with smoking, depressive symptoms, and anxiety. J Adolesc Health.

[38] Ward C, Kennedy A (1999). The measurement of sociocultural adaptation. Int J Intercult Relat.

[39] Cemalcilar Z, Falbo T, Stapleton LM (2005). Cyber communication: a new opportunity for international students’ adaptation?. Int J Intercult Relat.

[40] Hui L. A research of China higher education student satisfaction based on PLS-SEM model. 2011 (Doctoral dissertation). Jiangsu University. http://d.wanfangdata.com.cn/thesis/Y1895437. Accessed 24 Aug 2011.

[41] Cohen S, Kamarck T, Mermelstein R (1983). A global measure of perceived stress. J Health Soc Behav.

[42] Cohen S, Williamson G, Spacapam S, Oskamp S (1988). Perceived stress in a probability sample of the United States. The social psychology of health: Claremont Symposium on Applied Social Psychology.

[43] Buysse DJ, Reynolds CF, Monk TH, Berman SR, Kupfer DJ (1989). The Pittsburgh Sleep Quality Index: a new instrument for psychiatric practice and research. Psychiatry Res.

[44] Li Z, Liu Y (2018). Research methods for nursing.

[45] Oladosu FA, Tu FF, Farhan S, Garrison EF, Steiner ND, Roth GE (2018). Abdominal skeletal muscle activity precedes spontaneous menstrual cramping pain in primary dysmenorrhea. Am J Obstet Gynecol.

[46] Raval CM, Panchal BN, Tiwari DS, Vala AU, Bhatt RB (2016). Prevalence of premenstrual syndrome and premenstrual dysphoric disorder among college students of Bhavnagar, Gujarat. Indian J Psychiatry.

[47] Fernández-Martínez E, Dolores M, Abreu-Sánchez A, González-Sanz JD, Iglesias-López MT, Fernández-Muñoz JJ (2021). Menstrual migraine among Spanish university students. J Pediatr Nurs.

[48] Fernández MdM, Regueira-Méndez C, Takkouche B (2019). Psychological factors and premenstrual syndrome: a Spanish case-control study. PLoS ONE.

[49] Mohib A, Zafar A, Najam A, Tanveer H, Rehman R (2018). Premenstrual syndrome: existence, knowledge, and attitude among female university students in Karachi. Cureus.

[50] Gollenberg AL, Hediger ML, Mumford SL, Whitcomb BW, Hovey KM, Wactawski-Wende J (2010). Perceived stress and severity of perimenstrual symptoms: the BioCycle Study. J Womens Health.

[51] Liu Q, Wang Y, Van Heck CH, Qiao W (2017). Stress reactivity and emotion in premenstrual syndrome. Neuropsychiatr Dis Treat.

[52] Schliep KC, Mumford SL, Vladutiu CJ, Ahrens KA, Perkins NJ, Sjaarda LA (2015). Perceived stress, reproductive hormones, and ovulatory function: a prospective cohort study. Epidemiology.

[53] Schlarb AA, Friedrich A, Claßen M (2017). Sleep problems in university students—an intervention. Neuropsychiatr Dis Treat.

[54] Abdulah DM, Piro RS (2018). Sleep disorders as primary and secondary factors in relation with daily functioning in medical students. Ann Saudi Med.

[55] Arafa A, Mahmoud O, Salem EA, Mohamed A (2020). Association of sleep duration and insomnia with menstrual symptoms among young women in Upper Egypt. Middle East Curr Psychiatry.

[56] Lustig K, Stoakley E, MacDonald K, Geniole S, McCormick C, Cote K (2017). Sex hormones play a role in vulnerability to sleep loss on emotion processing tasks. Neurobiol Sleep Circad Rhythms.

[57] Yang P, Dervin F, Du X, Härkönen A (2018). Journey to the East: intercultural adaptation of international students in China. International students in China: education, student life and intercultural encounters.

